# Kinematic Decoupling Analysis and Design of a Biomimetic Robotic Elbow Joint

**DOI:** 10.1155/2018/4613230

**Published:** 2018-05-09

**Authors:** Bingyan Cui, Liwen Chen, Yongtao Xie, Zhijun Wang

**Affiliations:** ^1^College of Mechanical Engineering, North China University of Science and Technology, Tangshan 063009, China; ^2^Yanshan University, Qinhuangdao 066009, China; ^3^Widex Sanitary Co. Ltd., Tangshan 063000, China

## Abstract

The research of a biomimetic robotic manipulator is based on the flexible characteristics of the human upper limb joint, and a biomimetic robotic elbow joint plays a very significant role in the kinematic control of the biomimetic robotic manipulator. Most robotic elbow joints encountered today have a common disadvantage of bad neutrality, low rotational capability, and poor biomimetics. To overcome some difficulties, this paper presents a novel biomimetic robotic elbow joint. The structural model of the elbow joint is described, and the position equation is solved. Secondly, the kinematic equation of the elbow joint is established, the kinematic decoupling performance evaluation index of the elbow joint is defined, the kinematic decoupling characteristics of the elbow joint are analyzed, and the kinematic decoupling performance map in the workspace is drawn. Thirdly, using the spatial model theory, the structural parameters of the elbow joint are optimized, the structural parameters are selected by the Monte Carlo method, and the novel biomimetic robotic elbow joint is designed. The analysis results showing the kinematic decoupling performance of the elbow joint are symmetrical and the kinematic decoupling performance decreases with the increase of the angle, and there is a good kinematic decoupling in the workspace of about 35% in the vicinity of the initial position. When the structural parameters of the elbow joint are *R*_e1_ = 90 mm, *R*_e2_ = 70 mm, and *R*_e3_ = 30 mm, the elbow joint has a very good kinematic decoupling. This paper can lay a foundation for further analysis and research of the biomimetic robotic elbow joint.

## 1. Introduction

Biomimetic robotics is a new branch in the field of robot researches, which have integrated the biomimetic principle into the design and control of the robot and could imitate structure and movement characteristics of animals or humans. The motion behavior and some functions of natural organism have provided a great deal of thinking sources for robot scientists to design and realize flexible control [1–3]. With flexible operation and movement features, biomimetic robotics based on various types of bionic joints has highlighted good application prospects in the fields of rehabilitation medicine, space exploration, rescue, and so on [4–6].

The research of a biomimetic robotic manipulator is based on the flexible characteristics of the human upper limb joint and dexterous hands, and the human upper limbs are composed of the shoulder joint, elbow joint, wrist joint, and finger joint to complete the complex work and reflect the flexibility of the whole limb movement. The more flexible the upper limb joint, the more flexible the upper limb movement can be controlled [7]. Li et al. [8] designed a novel 3-DOF shoulder joint, established the error performance index, and plotted the error map. Li et al. [9] proposed a bionic eye based on a 3-DOF spherical parallel mechanism, and the structure parameters of the bionic eye were optimized. Klein et al. [10] designed a 3-DOF exoskeleton shoulder joint, and the torque capacity character curve is obtained. Zhang and Jin [11] made an in-depth study of theory on the driving characteristics, obtained dynamic characteristics, and optimized size of a 3-DOF shoulder joint. Sun et al. [12] proposed a wrist joint based on a spherical 3-DOF parallel decoupling mechanism, and the position of the wrist joint was analyzed.

Cui and Jin [13] presented a 2-DOF elbow joint, defined the static performance index, and obtained the static performance curve. In 2015, a novel hip joint is designed and its dynamic characters and structural parameters have studied [14]. Xu et al. [15] designed an elbow joint based on large deceleration ratio traction motor direct drive structure and analyzed the force character, and the elbow provided effective flexion and extension movement based on the double-screw-pair transmission and the elbow joint developed and completed flexion and extension movement [16]. Hwang et al. [17] proposed a novel elbow based on a slider-crank mechanism and obtained the force transmission property curve. Stanišić and Goehler [18] proposed a hybrid shoulder mechanism for copying the grasping movement, and a mechanism capable of reproducing voluntary human reaching motions is introduced along with the procedural method of implementing the coupled motions. Lovasz et al. [19] designed an elbow module, carried on remote control for a haptic arm exoskeleton, and proposed several design solutions and a control strategy. A biomimetic robotic elbow joint plays a very significant role in the motion control of a biomimetic robotic manipulator. The kinematic decoupling is an important index which affects the overall motion and the control of the biomimetic robotic elbow joint. The kinematic decoupling of the mechanism [20] can determine its kinematic characteristics and provide the basis for the robot control and trajectory planning.

The problem of decoupling is closely related to the Jacobian matrix of the biomimetic robotic joint. Researchers have studied less in this field. Gong et al. [21] proposed the decoupling of the moving coordinates in the absolute coordinates and verified the decoupling theory by the corresponding examples. Shen et al. [22] analyzed the kinematic decoupling of a 6-DOF parallel mechanism. Xu et al. [23] studied the 2R1T parallel mechanism on the principle of its motion decoupling based on the positional relationship between the actuation force and the rotational axis. Essomba and Nguyen Vu [24] presented a novel spherical decoupled mechanism and proved the decoupling motion by its kinematic and velocity models.

Based on the analysis of the literature, the existing robotic elbow joint is usually able to achieve flexion and extension. The structure of elbow joint design adopts a series structure; the series structure lacks good centering ability. From the analysis of the performance of elbow joints, there are many literatures on the bearing capacity of elbow joints, and the literature of the kinematic decoupling has not been seen yet. On this basis, this paper proposes a biomimetic robotic elbow joint. The biomimetic robotic elbow joint adopts a 2-DOF spherical parallel mechanism as a prototype which has advantages of good structural characteristics, large range of motion, and high bearing capacity and was compared with the 3-DOF parallel mechanism in the 863 project. From the analysis of the degree of freedom of the mechanism, the 3-DOF spherical parallel mechanism has three degrees of freedom in accordance with the requirements of the prosthetic prototype of the shoulder joint, and the human elbow joint structure has two degrees of freedom of movement characteristics, so the elbow joint mechanism has very good bionic structure. From the analysis of the installation of the mechanism, the rotation center of the 3-DOF spherical parallel mechanism is located in the middle of the moving platform and the static platform. The neutrality requirements for the rods during installation are very high and difficult to install. The rotation center of each rod of the biomimetic robotic elbow joint presents 90 degrees, which is convenient to install and has good neutrality.

In this paper, the kinematic equation of the biomimetic robotic elbow joint is established, the Jacobian matrix is derived, the kinematic decoupling performance evaluation index of the biomimetic robotic elbow joint is defined, the kinematic decoupling performance evaluation index map is drawn in the workspace, and the global kinematic decoupling performance index is established based on the kinematic decoupling analysis. Based on the space model theory, the structural parameters of the elbow joint are optimized and selected by the Monte Carlo method, and the biomimetic robotic elbow joint is designed. The purpose of the decoupling analysis of the elbow joint is to make the control of the elbow joint mechanism more convenient and easier.

## 2. The Model and Position Analysis

### 2.1. The Structural Description

**Figure 1:**
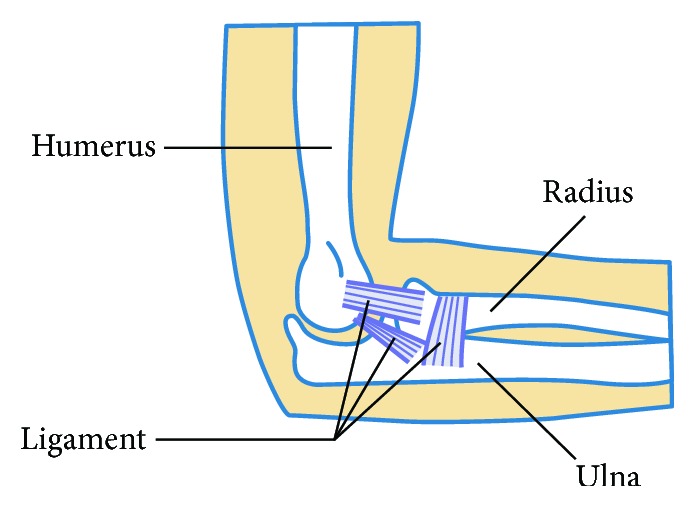
Schematic diagram of the human elbow joint.

The biomimetic robotic elbow joint is an important joint of the biomimetic hand. On the base of the knowledge of bionics, the human elbow joint is composed of the humerus, radius, ulna, and ligament. The human elbow joint is limited to the connection between the three bones and the ligament, which can only rotate about two axes and can realize movement of internal rotation and external rotation and flexion and extension, as shown in Figure 1.

**Figure 2:**
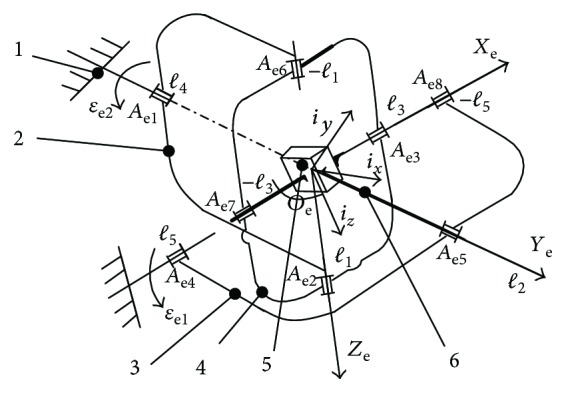
The structure of the biomimetic robotic elbow joint. 1—frame, 2 and 3—connecting rods, 4—annular member, 5—moving platform, and 6—arm connector.

The structure design of the biomimetic robotic elbow joint should be able to realize the movement of the anthropomorphic elbow joint. According to the analysis of joint structure and kinematic characteristics of the human elbow joint, this paper proposed a novel biomimetic robotic elbow joint based on a 2-DOF orthogonal spherical parallel mechanism. The elbow joint has two moving branches, the frame and the moving platform, which contain two driving motors, and the driving motor is fixed on the frame. The moving platform of the elbow joint is connected by the arm connector and connected with the ring through the rotating pair. The elbow joint can realize two degrees of freedom of rotation and has advantages of good bionic characteristics, small structure, and little inertial forces. The structure diagram is shown in Figure 2.

Two Cartesian coordinates, the base frame {*C*_e_} is fixed and represented by the coordinate [*X*_e_ *Y*_e_ *Z*_e_], while the moving platform has a frame, {*K*_e_}, attached to it with coordinates [*i*_*x*_*i*_*y*_*i*_*z*_]. The coordinate center of the two coordinate systems is coincident, and the center point of coordinates is *O*_e_. The plane *A*_*o*_ is composed of *O*_e_*A*_e1_ and *O*_e_*A*_e4_ axes. The *X*_e_ axis is along *O*_e_*A*_e8_ the vector direction, the *Y*_e_ axis is along *O*_e_*A*_e5_ the vector direction and passes, and the *Z*_e_ axis is vertical to the plane *A*_*o*_ and along *O*_e_*A*_e2_ the vector direction. **ℓ**_1_ is the unit vector of *O*_e_*A*_e2_ axes, **ℓ**_2_ is the unit vector of *O*_e_*A*_e5_ axes, **ℓ**_3_ is the unit vector along *O*_e_*A*_e3_, **ℓ**_4_ is the unit vector of *O*_e_*A*_e1_ axes, and **ℓ**_5_ is the unit vector of *O*_e_*A*_e4_ axes.

Assume that the spherical radius of the reference center of the connection rotation pairs *A*_e1_ and *A*_e4_ is *R*_e1_, the spherical radius of the reference center of the connection rotation pairs *A*_e3_ and *A*_e5_ is *R*_e2_, and the spherical radius of the reference center of the connection rotation pairs *A*_e2_ is *R*_e3_. Therefore, the reference centers of the rotation pairs are distributed on concentric spherical surfaces with different radii, *R*_e3_ < *R*_e2_ < *R*_e1_. Thus, the structural parameters of the elbow joint are *R*_e1_, *R*_e2_, and *R*_e3_.

### 2.2. Derivation of Position Forward Solution

The kinematic equation of the elbow joint is established based on the solution of the inverse position. The attitude angles of the elbow joint moving platform are *γ*_e_ and *β*_e_, and the input angles are *γ*_e_ and *β*_e_. The moving platform of the elbow joint can rotate around the *X* axis and the *Y* axis, and the corresponding transformation matrix of the elbow joint moving platform is introduced and can be written as

(1) $$\begin{array}{c} T_{e}=T\left(X,\gamma_{e}\right)T\left(Y,\beta_{e}\right)=\left[\begin{array}{ccc} \cos\beta_{e} & 0 & \sin\beta_{e}\\ \sin\gamma_{e}\sin\beta_{e} & \cos\gamma_{e} & -\sin\gamma_{e}\cos\beta_{e}\\ -\cos\gamma_{e}\sin\beta_{e} & \sin\gamma_{e} & \cos\gamma_{e}\cos\beta_{e} \end{array}\right]. \end{array}$$

In the fixed coordinate system, the unit vector **ℓ**_2_ can be expressed as

(2) $$\begin{array}{c} \ell_{2}=T_{e}\;\left(\begin{array}{c} 0\\ 1\\ 0 \end{array}\right)=\left(\begin{array}{c} 0\\ \cos\gamma_{e}\\ \sin\gamma_{e} \end{array}\right). \end{array}$$

According to the mechanism characteristics of the elbow joint, the input angle of the drive motor is *ε*_e1_ and the unit vector **ℓ**_2_ can be expressed as the input angle *ε*_e1_ and is given by

(3) $$\begin{array}{c} \ell_{2}=\left(\begin{array}{ccc} 1 & 0 & 0\\ 0 & \cos\varepsilon_{e1\;} & -\sin\varepsilon_{e1}\\ 0 & \sin\varepsilon_{e1} & \cos\varepsilon_{e1} \end{array}\right)\left(\begin{array}{c} 0\\ 1\\ 0 \end{array}\right)=\left(\begin{array}{l} 0\\ \cos\varepsilon_{e1}\\ \sin\varepsilon_{e1} \end{array}\right). \end{array}$$

Comparison of (2) and (3) can be obtained as

(4) $$\begin{array}{c} \varepsilon_{e1}=\gamma_{e}. \end{array}$$

In the fixed coordinate system, the unit vector **ℓ**_3_ can be expressed as

(5) $$\begin{array}{c} \ell_{3}=T_{e}\left(\begin{array}{c} 1\\ 0\\ 0 \end{array}\right)=\left(\begin{array}{c} \cos\beta_{e}\\ \sin\gamma_{e}\sin\beta_{e}\\ -\sin\gamma_{e}\cos\beta_{e} \end{array}\right). \end{array}$$

In the fixed coordinate system, the unit vector **ℓ**_1_ is represented by the input angle *ε*_e2_ and is given by

(6) $$\begin{array}{c} \ell_{1}=\left[\begin{array}{ccc} \cos\varepsilon_{e2} & 0 & \sin\varepsilon_{e2}\\ 0 & 1 & 0\\ -\sin\varepsilon_{e2} & 0 & \cos\varepsilon_{e2} \end{array}\right]\left(\begin{array}{c} 0\\ 0\\ 1 \end{array}\right)=\left(\begin{array}{c} \sin\varepsilon_{e2}\\ 0\\ \cos\varepsilon_{e2} \end{array}\right). \end{array}$$

According to the geometry of the elbow joint, (7) can be obtained.

(7) $$\begin{array}{c} \ell_{1}\cdot \ell_{3}=0. \end{array}$$

Equations (2) and (3) can be written in the form of (7) and can be derived as

(8) $$\begin{array}{c} \varepsilon_{e2}=a\tan2\left(\cos\gamma_{e}\sin\beta_{e},\cos\beta_{e}\right). \end{array}$$

The inverse position solution equation of the elbow joint can be written as

(9) $$\begin{array}{c} \varepsilon_{e1}=\gamma_{e},\\ \varepsilon_{e2}=a\tan2\left(\cos\gamma_{e}\sin\beta_{e},\cos\beta_{e}\right), \end{array}$$

The forward positive solution equation of the elbow joint by (9) is deduced as

(10) $$\begin{array}{c} \gamma_{e}=\varepsilon_{e1},\\ \beta_{e}=a\tan2\left(\sin\varepsilon_{e2},\cos\varepsilon_{e1}\cos\varepsilon_{e2}\right). \end{array}$$

## 3. The Kinematic Decoupling Analysis

### 3.1. The Derivation of the Kinematic Equation

Differential treatment (10), the kinematic equation is obtained as

(11) $$\begin{array}{c} {\dot{\phi }}_{e}=J_{e}{\dot{\varepsilon }}_{e}, \end{array}$$

where ${\dot{\phi }}_{e}$ is the output angle velocity and is expressed as ${\dot{\phi }}_{e}={\left[{\dot{\gamma }}_{e}\;{\dot{\beta }}_{e}\right]}^{T}$, ${\dot{\varepsilon }}_{e}$ is the input angle velocity and is expressed as ${\dot{\varepsilon }}_{e}={\left[\begin{array}{cc} {\dot{\varepsilon }}_{e1} & {\dot{\varepsilon }}_{e1} \end{array}\right]}^{T}$, and **J**_e_ is the Jacobian matrix and can be extracted as

(12) $$\begin{array}{c} J_{e}=\left(\begin{array}{cc} 1 & 0\\ \frac{\sin\varepsilon_{e1}\sin\varepsilon_{e2}\cos\varepsilon_{e2}}{\sin^{2}\varepsilon_{e2}+\cos^{2}\varepsilon_{e1}\cos^{2}\varepsilon_{e2}} & \frac{\cos\varepsilon_{e1}}{\sin^{2}\varepsilon_{e2}+\cos^{2}\varepsilon_{e1}\cos^{2}\varepsilon_{e2}} \end{array}\right). \end{array}$$

The kinematic decoupling analysis of the elbow joint depends on the Jacobian matrix **J**_e_. In order to describe the decoupling characteristic in more detail, the index of kinematic decoupling performance is defined as

(13) $$\begin{array}{c} {℘}_{e}=\frac{\varsigma_{e\max}-\varsigma_{e\min}}{\varsigma_{e\max}}, \end{array}$$

where the maximum and minimum singular values of the elbow joint Jacobian matrix are *ς*_emax_ and *ς*_emin_, respectively.

### 3.2. Analysis of Kinematic Decoupling Performance

The decoupling analysis of the kinematic of the elbow joint is to study the correlation or dependence of the output parameters on the input parameters. If the Jacobian matrix **J**_e_ can be turned into a diagonal matrix, the elbow joint is kinematic decoupling. The kinematic decoupling of the elbow joint under different input conditions is analyzed.

**Figure 3:**
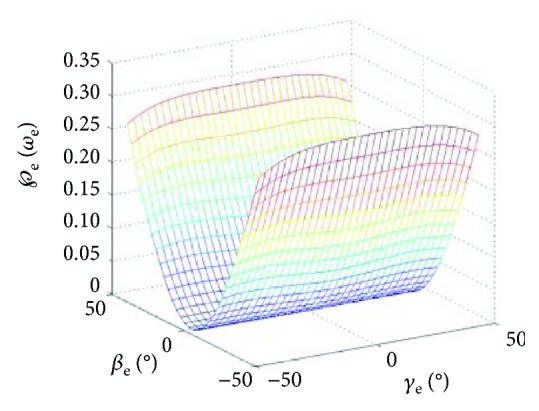
Index of the kinematic decoupling performance of output angle velocity when the input velocity has the *X*_e_ axis and *Y*_e_ axis.

(1) When the input angular velocity of the *X*_e_ axis is ${\dot{\varepsilon }}_{e1}$, the decoupling of the output angular velocity of the elbow joint is analyzed.
The elbow joint has an input angular velocity on the *X*_e_ axis, and the input angular velocity on the *Y*_e_ axis is ${\dot{\varepsilon }}_{e2}$ and ${\dot{\varepsilon }}_{e2}=0\;rad/s\;$. The Jacobian matrix of the elbow joint can be obtained from (12) and can be given by
  (14) $$\begin{array}{c} J_{e}=\left(\begin{array}{cc} 1 & 0\\ 0 & 1 \end{array}\right). \end{array}$$
From (14), the Jacobian matrix is a diagonal unit matrix and the evaluation index of the kinematic decoupling performance in the workspace is always zero, so the elbow joint is completely kinematic decoupling and has the highest decoupling performance.
(2) When the input angular velocities of the *X*_e_ axis and *Y*_*e*_ axis are ${\dot{\varepsilon }}_{e1}$ and ${\dot{\varepsilon }}_{e2}$, the elbow joint kinematic decoupling is analyzed.
When the elbow joint is driven by two driving motors, (12) expresses the Jacobian matrix. With the decoupling definition of the local motion of the shoulder joint, there is only one nonzero element in the first row of (12), so the elbow joint is locally kinematic decoupling in the whole workspace. According to the kinematic decoupling index of (13), the local kinematic decoupling performance graph of the elbow joint is obtained, as shown in Figure 3.

  In Figure 3, the kinematic decoupling index ℘_e_ changes little with the increase of the *γ*_e_ angle. With the increase of the *β*_e_ angle, the kinematic decoupling index ℘_e_ increases gradually, its decoupling becomes smaller, and the coupling gradually increases. The kinematic decoupling of the elbow joint is locally decoupling in the *γ*_e_ angle direction, with very good kinematic decoupling in the region of approximately 35% of the initial position.

## 4. Global Kinematic Decoupling Analysis and Structural Parameter Optimization

The global performance index can better reflect the kinematic decoupling of the elbow joint in the whole posture workspace, and the spatial model theory [25, 26] provides a new idea for the structural parameter optimization of the elbow joint. The structural parameters of the elbow joint are optimized by the theory of the space model, which made the performance evaluation index of kinematic decoupling better. The structure parameters of the elbow joint were selected by the Monte Carlo method.

### 4.1. Analysis of the Global Kinematic Decoupling Performance

Based on the structural description of the biomimetic robotic elbow joint, the structural parameters are *R*_e1_, *R*_e2_, and *R*_e3_. The spatial model of the structural parameters of the biomimetic robotic elbow joint is established. The structural dimension parameters of the elbow joint are dimensionless and can be given as

(15) $$\begin{array}{c} l_{e}=\frac{R_{e1}+R_{e2}+R_{e3}}{3}. \end{array}$$

The dimensionless structural dimensions of the elbow joint are expressed, respectively, as

(16) $$\begin{array}{c} l_{e1}=\frac{R_{e1}}{l_{e}},\\ l_{e2}=\frac{R_{e2}}{l_{e}},\\ l_{e3}=\frac{R_{e3}}{l_{e}}. \end{array}$$

Formula (16) can be obtained as

(17) $$\begin{array}{c} l_{e1}+l_{e2}+l_{e3}=3. \end{array}$$

Considering the manufacturing and assembling of the biomimetic robotic elbow joint, the conditions for the structural parameters are satisfied and can be written as

(18) $$\begin{array}{c} 0\leq l_{e3}\leq l_{e2}\leq 3,\\ 0\leq l_{e1}\leq 3. \end{array}$$

**Figure 4:**
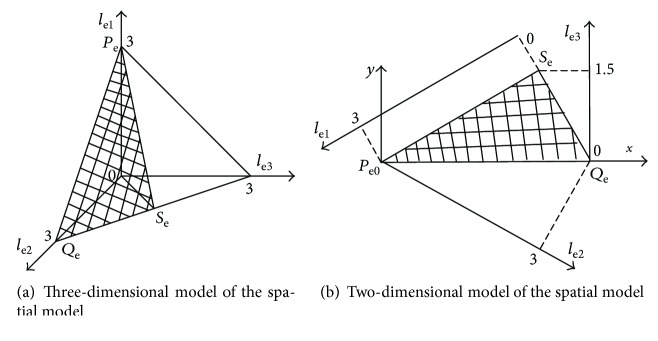
The spatial model of the biomimetic robotic elbow joint.

The three dimensionless structure parameters are Cartesian axes *l*_e1_, *l*_e2_, and *l*_e3_, respectively. The geometric space model of the biomimetic robotic elbow joint and the triangle Δ*P*_e_*Q*_e_*S*_e_ can be obtained by using (15) and (18), as shown in Figure 4. In order to facilitate drawing, the three-dimensional model is transformed into a two-dimensional model of the space model. The transformation relation of the dimensionless coordinate is generated as

(19) $$\begin{array}{c} x=\frac{\sqrt{3}+\left(l_{e2}-l_{e1}\right)}{\sqrt{3}},\\ y=l_{e3}. \end{array}$$

The global performance index is introduced into the spatial model, and the global kinematic decoupling performance index of the biomimetic robotic elbow joint is defined as

(20) $$\begin{array}{c} \xi_{e}=\frac{\int_{V_{e}}{℘}_{e}{dV}_{e}}{\int_{V_{e}}{dV}_{e}}, \end{array}$$

where *ξ*_e_ is the global kinematic decoupling performance index and *V*_e_ is the workspace of the elbow joint.

**Figure 5:**
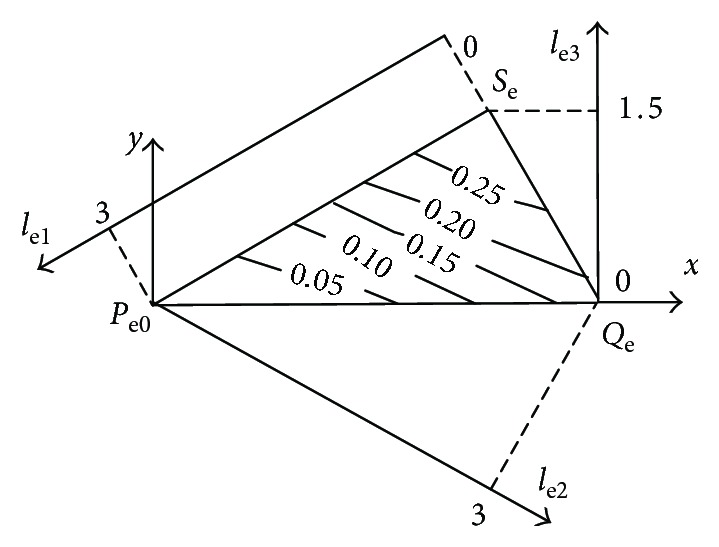
Globe kinematic decoupling performance evaluation index atlases of the elbow joint.

Using MATLAB software, the global kinematic decoupling performance map of the elbow joint in the plain geometric space model is drawn according to (1), (2), (3), (4), (5), (6), (7), (8), (9), (10), (11), (12), (13), (14), (15), (16), (17), (18), (19), and (20), as shown in Figure 5. In Figure 5, according to (16), the three coordinates *l*_e1_, *l*_e2_, and *l*_e3_ reflect the three structural parameters *R*_e1_, *R*_e2_, and *R*_e3_, respectively.

From Figure 5, the global kinematic decoupling performance of the elbow joint is linearly distributed. With the increase of *R*_e3_ and *R*_e2_, the global kinematic decoupling performance index values increase and the global kinematic decoupling decreases gradually. With the increase of *R*_e1_, the global kinematic decoupling performance index values decrease and the global kinematic decoupling increases.

### 4.2. The Structural Parameter Optimization

Structural parameters have a direct influence on the kinematic performance of the robot. Stan et al. [27] defined the optimal function based on stiffness and transmission quality. In this paper, the range of structural parameters of the elbow joint is defined. For the optimal design of the elbow joint, reasonable structural parameters should be selected for design and manufacturing. The analysis of global kinematic decoupling performance of the elbow joint based on the spatial model theory has laid the theoretical foundation for the selection of the elbow joint structural parameters. In this paper, based on the Monte Carlo method, the structural parameters of the elbow joint are optimized. The reasonable structural parameters of the elbow joint are obtained by using the probability statistic method.

The value range of the global kinematic decoupling performance index of the elbow joint is the interval [0, 1], which conforms to the rule of rectangle distribution. Combined with the structural features of the elbow joint, the structural parameter ranges of the elbow joint *R*_e1_, *R*_e2_, and *R*_e3_ are *R*_e1_ ∈ [60, 90] mm, *R*_e2_ ∈ [40, 70] mm, and *R*_e3_ ∈ [20, 50] mm.

In [28], the structural parameters of the mechanical arm are optimized using the Monte Carlo method, and the intermediate value of the performance index is selected as the structural parameter optimization objective. Therefore, the intermedia value of the global kinematic decoupling index of the elbow joint is used as the optimal target of the elbow joint, in which *ξ*_e_ = 0.1524. When *ξ*_e_ is not more than 0.1524, the global kinematic decoupling performance is better.

**Figure 6:**
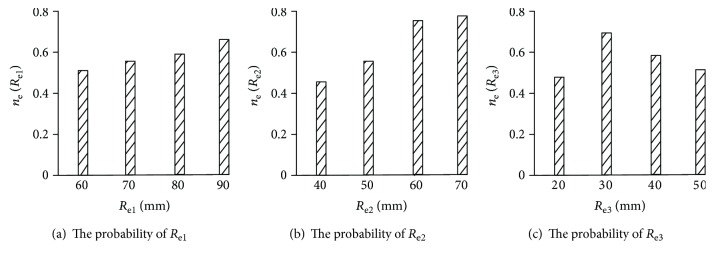
Probability distribution of the discrete histogram for the structure parameters for the biomimetic robotic elbow joint.

Under the condition that the evaluation index of the global kinematic decoupling performance is better than the optimization target value, the distribution rule of sampling values of each parameter counted the sampling within the range of structural parameters, and the probability distribution of the structural parameters is obtained, as shown in Figure 6. It is clear from Figure 6 that the structure parameters are *R*_e1_ = 90 mm, *R*_e2_ = 70 mm, and *R*_e3_ = 30 mm, and the probability model of the structural parameters of the biomimetic robotic elbow joint is higher.

## 5. The Design of the Biomimetic Robotic Elbow Joint

Considering its processing and assembling process, the virtual prototype of the elbow joint is designed based on the optimized structure parameters, and primary technical parameters are shown in Table 1.

**Figure 7:**
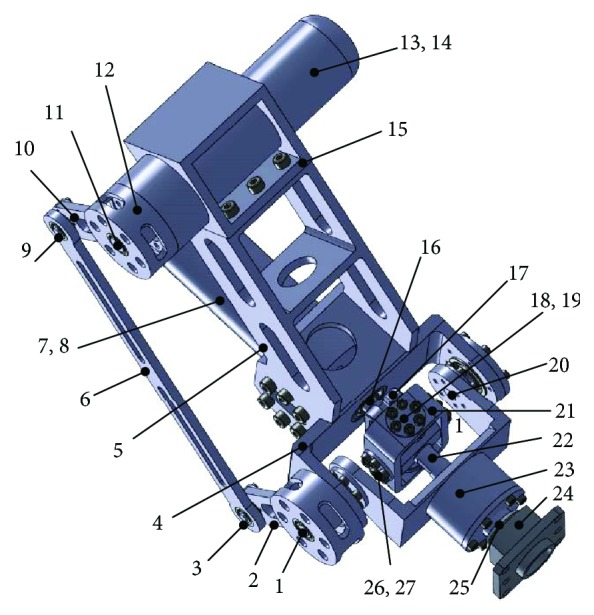
A novel biomimetic robotic elbow joint virtual prototype. 1, 3, 9, 11, 16, 18, 19, 20, 25, 26, and 27—revolute joints; 2 and 10—short bars; 4—frame; 5—upper arm; 6—long bar; 7 and 13—motor; 8 and 14—connectors; 12–15—motor bases; 17 and 23—connecting rods; 21—annular member; 22—moving platform; and 24—arm connector.

The virtual prototype model of the biomimetic robotic elbow joint is shown in Figure 7.

Servo motor 13 is installed on motor base 15, and servo motor 13 transmits the torque of the motor to carrier rod 23 through the planar four-bar mechanism. The other movement branch of the biomimetic robotic elbow joint is servo motor 7 mounted on elbow base 5. Servo motor 7 is fixedly connected with the rotating shaft on the bottom of connecting rod 17 through the mounting hole of coupling 8. Connecting rod 17 is connected to annular member 21 by a pair of coaxial rotating hinges 18 and 19. The other pair of coaxial rotary hinges 26 and 27 on annular member 21 is connected to moving platform 22. Arm connector 24 is fixedly connected to the bottom of the moving platform.

## 6. Simulation Experimental Analyses

### 6.1. Kinematic Simulation Experiment

In order to verify the correctness of the kinematic equations of the elbow joint mechanism, the results of the theoretical solution of the elbow joint and the simulation of the kinematic model are compared and analyzed. The movement trajectory of the biomimetic robotic elbow joint moving platform is expressed by

(21) $$\begin{array}{c} \gamma_{e}=\sin\left(t\right)\left(rad\right),\\ \beta_{e}=\cos\left(t\right)\left(rad\right). \end{array}$$

**Figure 8:**
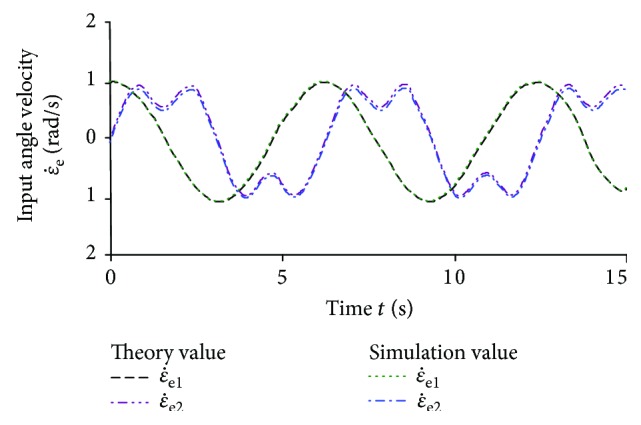
The input angle velocity curve of the elbow joint.

According to (11), under a given movement trajectory, the theoretical input angular velocity curve can be obtained by using MATLAB, as shown in Figure 8. A virtual prototype of the elbow joint is designed based on the optimized structure parameters, and the simulation value of the movement change curve of the input angle of the virtual prototype is obtained by using ADAMS movement simulation software, as shown in Figure 8. From Figure 8, the theoretical values and the simulation values are almost identical, which verifies the correctness of the modeling of the movement equation.

### 6.2. Kinematic Decoupling Simulation Experiment

A virtual prototype of the biomimetic robotic elbow joint is established, and a virtual simulation experiment is performed. Based on the Core software, the elbow joint is parametric designed. The parameter design range is the same as the parameter range in the spatial model theory. The parametric design variable increment is 5 mm, and 343 virtual prototypes of elbow joints with different structural dimensions were obtained.

The kinematic simulation of the reachable workspace is carried out for each prototype, and the total volume number of workspaces and the number volume of workspaces conforming to the kinematic decoupling index are recorded.

The structural parameters were obtained by optimizing the spatial model, which are 30 mm, 70 mm, and 90 mm. The kinematic decoupling workspace volume number is taken as the standard value *W*_d0_, and the virtual prototype is defined by the standard prototype. The volume number of kinematic decoupling workspaces obtained by other different structural parameters is *W*_d_. The kinematic decoupling workspace volume ratio Wds is defined as

(22) $$\begin{array}{c} Wds=\frac{W_{d}}{W_{d0}}\times 100\%. \end{array}$$

**Figure 9:**
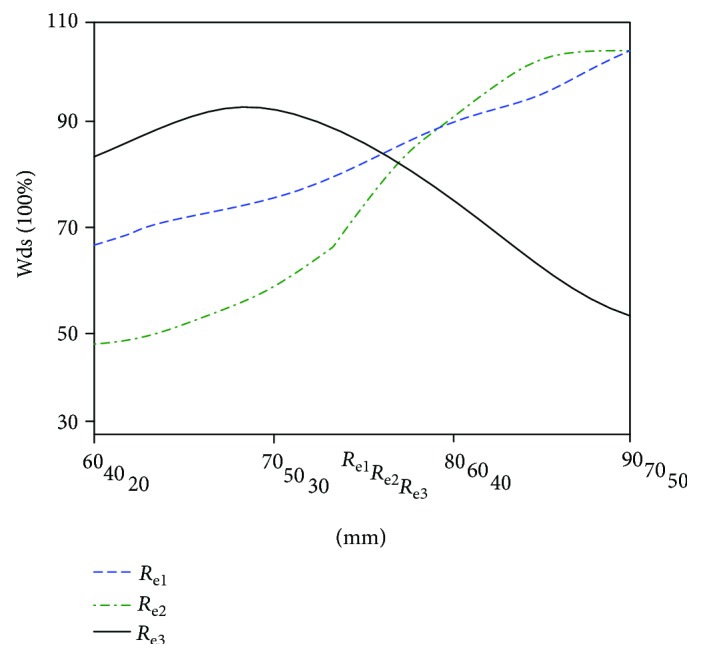
The influence curve of structural parameters on the kinematic decoupling workspace volume ratio.

The 343 elbow joint movement decoupling volume numbers are compared with the basic kinematic decoupling volume number. When one structural parameter is changed and the other parameters are unchanged in the standard prototype, the volume number of kinematic decoupling workspaces is acquired, and the influence curve of each structural parameter on the size of the elbow joint kinematic decoupling workspace is drawn, as shown in Figure 9.

In Figure 9, the kinematic decoupling volume ratio Wds increases with the increase of *R*_e2_ and *R*_e3_, and with the increase of *R*_e3_, the volume ratio of the kinematic decoupling workspace firstly increases and then decreases. The change trend is the same as that in Figure 6, the rationality of optimization of the structural parameters can be proved, and the kinematic decoupling analysis is correct.

## 7. Conclusion

The kinematic decoupling analysis of a novel biomimetic robotic elbow joint was performed. Analysis of the kinematic decoupling of the elbow joint is symmetrical in the workspace, and the motion is completely decoupling when driven by a single motor. The global kinematic decoupling performance of the elbow joint showed a linear distribution. With the increase of *R*_e3_, the decoupling decreased gradually. The structure parameters of the elbow joint were selected by the Monte Carlo method, which are*R*_e1_ = 90 mm, *R*_e2_ = 70 mm, and *R*_e3_ = 30 mm. Considering the process and the assembly process, the elbow joint prototype had been designed and developed. Finally, simulation experiments have verified the correctness of the kinematic equation and kinematic decoupling, as well as the rationality of the optimization use of spatial model parameters.

In summary, the biomimetic robotic elbow joint has two degrees of freedom and good decoupling characteristics, which can be applied in rehabilitation robot, biomimetic robot, industrial robot, humanoid robot arm, and other fields.

## Figures and Tables

**Table 1:** The primary technical parameters of the biomimetic robotic elbow joint.

Design parameter	Technical index
Degree of freedom	2
Flexion	45°
Extension	45°
Internal rotation	50°
External rotation	45°
*R*_e1_	90 mm
*R*_e2_	70 mm
*R*_e3_	30 mm
Motor	Maxon 273755
Coupling	Maxon 326665
Encoder	MR TL 256-1024 CPT
